# Effect of Psychosocial Factors on Cancer Risk and Survival

**DOI:** 10.2188/jea.JE20130124

**Published:** 2014-01-05

**Authors:** Naoki Nakaya

**Affiliations:** Division of Personalized Prevention and Epidemiology, Department of Preventive Medicine and Epidemiology, Tohoku Medical Megabank Organization, Tohoku University, Sendai, Japan; 東北大学東北メディカル・メガバンク機構予防医学・疫学部門

**Keywords:** cancer, cohort study, depression, personality, psychosocial factors, risk, survival

## Abstract

Psychosocial factors such as personality traits and depression may alter immune and endocrine function, with possible effects on cancer incidence and survival. Although these factors have been extensively studied as risk and prognostic factors for cancer, the associations remain unclear. The author used data from prospective cohort studies in population-based and clinical databases to investigate these relations. The findings do not support the hypotheses that personality traits and depression are direct risk factors for cancer and cancer survival.

Some researchers have recently reported that cancer affects the psychological status of the partners and family members of cancer patients. The mechanisms underlying this hypothesis imply the existence of not only psychological distress from caregiving and grief but also a shared unhealthy lifestyle. Only a few studies have suggested that major psychosocial problems develop in partners of cancer patients. The present study used nationwide population-based data to investigate depression risk among male partners of women with breast cancer. The results support the hypothesis that such men are at increased risk of depression.

In conclusion, the effects of personality traits and depression on cancer risk and survival appear to be extremely small. In addition, partners of cancer patients were at increased risk of depression. Screening partners and family members of cancer patients for depressive symptoms is therefore an important concern for research in psycho-oncology.

## Abstract (ja)

パーソナリティ、抑うつなどの心理社会的要因は、免疫機能・内分泌機能を変化させ、がんの発症やがん予後に影響する可能性が指摘されている。これら心理社会的要因とがんの発症やがん予後の関連は多く報告されているが、未だ結論は得られていない。著者らは、一般地域住民を対象としたデータベース及び臨床データベースを用いて上記の関連を検討した。結果として、パーソナリティ、抑うつなどの心理社会的要因ががんの発症やがん予後と関連せず、仮説は否定された。

一方、最近の報告において、がん患者のみならずその配偶者・家族の健康影響が指摘されている。配偶者・家族の健康影響のメカニズムとして、がん患者への介護や死別による心理的・身体的負担、がん患者と共通する不健康な生活習慣が考えられる。先行研究において、がん患者の配偶者は重大な心理社会的問題を生じることが報告されているが、研究デザイン・研究規模などの問題があった。著者らは、デンマークの国家規模のデータベースを利用して、乳がん患者の男性パートナーのうつ病リスクを後ろ向きコホート調査にて検証した。結果として、男性パートナーのうつ病リスクは有意に増大し、仮説は支持された。

これまでの著者らの研究による結論として、パーソナリティ、抑うつなどの心理社会的要因とがんの発症やがん予後の関連はない、あるいは極めて小さいことが示された。一方、がん患者のパートナーのうつ病リスクは増大することが示された。今後、サイコオンコロジー分野において、がん患者のパートナー・家族の抑うつ症状のスクリーニングを実施し、早期発見することの重要性が明らかとなった。

## PERSONALITY TRAITS AND CANCER RISK

Personality traits have long been hypothesized to have a causal role in cancer development and progression. In 1962, Kissen and Eysenck conducted one of the first modern studies on the association between personality traits and cancer and reported that, as compared with hospital controls, patients with lung cancer were more likely to be extraverted and less likely to be neurotic,^1^ which could be interpreted to indicate that extraverts are at increased risk of cancer because they seek stimulation and thus experience high levels of stress, whereas individuals with low levels of neuroticism could be at increased risk of cancer because they tend to have fewer emotional outlets and therefore accumulate emotional stress.^2^ Greater exposure to stress could affect cancer risk by influencing immune and endocrine function.^3^^,^^4^

Since then, several well-conducted prospective studies found no association between personality traits (eg, extraversion, neuroticism, and trait anxiety) and cancer risk. However, most of these studies had methodologic limitations, including small numbers of incident cancers, which limited the statistical power to analyze site-specific cancer.

The present study used data from 2 large prospective cohort studies (the Miyagi cohort study^5^ and Swedish twin cohort/Finnish twin cohort^6^) to investigate the association between personality traits and cancer risk.

From June through August 1990, 30 277 residents of Miyagi Prefecture, in northern Japan, completed a Japanese version of the short form of the Eysenck Personality Questionnaire–Revised (EPQ-R) and a questionnaire on health habits.^5^ There were 671 prevalent cases of cancer at baseline, and 986 incident cases of cancer were identified during the 7-year follow-up, through December 1997. Multivariable hazard ratios (HRs) for total cancer among individuals in the highest versus the lowest quartile for a personality trait subscale were 0.9 for extraversion (95% CI = 0.7–1.1; *P* for linear trend = 0.32) and 1.2 for neuroticism (95% CI = 1.0–1.4; *P* for linear trend = 0.06) (Table 1).

**Table 1.** Multivariate-adjusted hazard ratios (HRs) and 95% CIs for cancer risk according to score quartile of personality trait subscale

Miyagi cohort study^5^ Outcome: Total cancer incidence	EPQ-R subscales	EPQ-R, Extraversion				EPQ-R, Neuroticism			
		
	Score group	Q1 (low)	Q2	Q3	Q4 (high)	Q1 (low)	Q2	Q3	Q4 (high)
		
	Multivariate HR (95% CI)	1.0 (Ref)	0.8 (0.7–1.0)	0.9 (0.8–1.1)	0.9 (0.7–1.1)	1.0 (Ref)	1.0 (0.8–1.2)	1.0 (0.9–1.2)	1.2 (1.0–1.4)
		
	*P* for linear trend	0.32				0.06			
	
	Covariates	Age, sex, smoking, alcohol, body mass index, education, family history of cancer							

Swedish/Finnish twin cohort^6^ Outcome: Total cancer incidence	EPI subscales	EPI, Extraversion				EPI, Neuroticism			
		
	Score group	Continuous variables				Continuous variables			
		
	Multivariate HR (95% CI)	0.99 (0.98–1.01)				1.00 (0.99–1.02)			
		
	*P* for linear trend	0.23				0.48			
	
	Covariates	Age, sex, smoking, alcohol, body mass index, education							

Abbreviations: EPG-R, Eysenck Personality Questionnaire–Revised; EPI, Eysenck Personality Inventory; Q, quartile.

Scores on personality subscales were divided into 4 categories to yield quartiles.

Additional analyses examined how study design (retrospective or prospective) and duration of follow-up (in prospective analyses) affected associations between personality scales and risk of total cancer. In the retrospective analysis, the 671 cancer cases at baseline, which had been ascertained from self-reports in the health-habit questionnaire or from cancer registry records, were used as an endpoint. Unconditional logistic regression was used to estimate odds ratios for the presence of prevalent cancers in relation to quartile of personality subscale scores. In the prospective analyses, 2 durations of follow-up were used: the first analysis encompassed the first 3 years of follow-up after the baseline, and the second encompassed 7 years of follow-up but excluded cancer cases diagnosed within the first 3 years. In analyses that examined the effect of study design (ie, retrospective vs prospective) and duration of follow-up (in the prospective study) on associations between personality subscales and risk of total cancer, the association with neuroticism differed depending on the type of analysis performed (Figure). Retrospective analysis (OR) showed a significant positive linear association between neuroticism and presence of cancer at baseline (*P* for linear trend < 0.001). Prospective analysis with only 3 years of follow-up (HR1) showed a significant positive linear association between neuroticism and the HR for incident cancer (*P* for linear trend = 0.03). However, in the second prospective analysis, which considered individuals during a follow-up period of 7 years but excluded cancer cases diagnosed in the first 3 years of follow-up (HR2), neuroticism was not associated with risk of incident cancer (*P* for linear trend = 0.43). These findings do not support the hypothesis that personality traits are a risk factor for cancer incidence. The association between neuroticism and prevalent cancer may be a consequence rather than a cause of cancer diagnosis and symptoms.^5^

**Figure.**
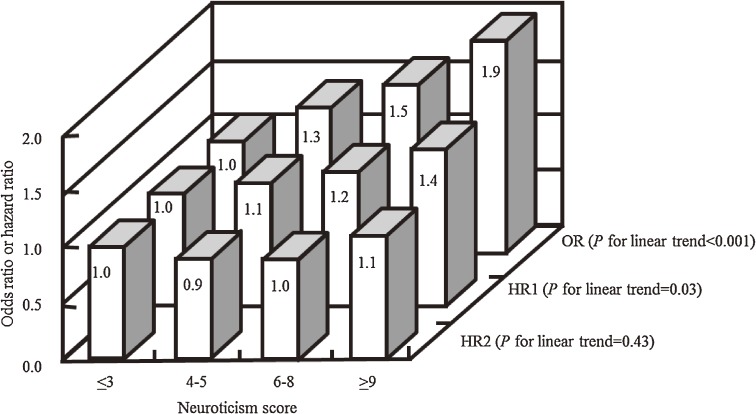
Association between neuroticism and overall cancer risk, according to study design (retrospective or prospective) and duration of follow-up (in prospective analyses).^5^ Cut-off points for neuroticism score were chosen so as to divide the population into 4 groups of similar size. In all analyses, the referent group was the group with the lowest neuroticism scores. OR denotes an odds ratio estimated from retrospective analysis of 671 prevalent cases of cancer at the baseline as the endpoint (*P* for linear trend < 0.001). HR1 denotes a hazard ratio estimated from prospective analysis of 320 incident cases of cancer diagnosed in the first 3 years of follow-up as the endpoint (*P* for linear trend = 0.03). HR2 denotes a hazard ratio estimated from prospective analysis of 666 incident cases of cancer diagnosed in years 4–7 of follow-up —ie, excluding cancer cases diagnosed in the first 3 years of follow-up— as the endpoint (*P* for linear trend = 0.43). All ORs and HRs were adjusted for sex, age, cigarette smoking (never smoker, past smoker, currently smoking 1–19 cigarettes per day, or currently smoking ≥20 cigarettes per day), alcohol consumption (never drinker, past drinker, currently drinking ≤22.7 g of alcohol per day, or currently drinking ≥22.8 g of alcohol per day), body mass index (≤18.4, 18.5–24.9, or ≥25.0 kg/m^2^), education (in school until age 15 years, 16–18 years, or ≥19 years), and family history of cancer (presence or absence in first-degree relatives).

The second prospective population-based cohort study comprised 59 548 Swedish (1974–1999) and Finnish (1976–2004) participants, who completed a questionnaire that included the Eysenck Personality Inventory (EPI) and items on health behavior at baseline.^6^ To analyze the association of extraversion and neuroticism with cancer risk, 4631 cancer cases were identified during a maximum follow-up period of 30 years. The present author used Cox proportional hazards models to estimate HRs for incidence of any cancer. HRs were estimated by treating personality trait subscale scores as continuous variables and are presented as risk per 1-unit increase in score for each scale. In multivariate analyses, extraversion and neuroticism were not significantly associated with overall cancer risk: the HRs were 0.99 for extraversion (95% CI = 0.9–1.01; *P* for linear trend = 0.23) and 1.00 for neuroticism (95% CI = 0.99–1.02; *P* for linear trend = 0.48) (Table 1). To the author’s knowledge, this is the largest study (>4500 incident cases) of the associations between personality traits and cancer risk. The findings are in line with those of recent prospective studies, which do not support the hypothesis that personality traits are direct risk factors for overall cancer risk.

## PERSONALITY TRAITS AND SURVIVAL AFTER CANCER

It as been suggested that personality traits have a role in cancer progression. Temoshok et al observed that tumor thickness in patients with malignant melanoma was positively associated with a “type C” personality, which they described as cooperative, unassertive, patient, suppressive of negative emotions, and accepting/compliant with external authorities.^7^ Patients with low extraversion and high neuroticism are believed to repress their emotions, which is considered one of the most important aspects of the type C personality.^8^ The hypothesis regarding cancer survival could also be interpreted as being related to stress. Accumulated repression of emotions may cause stress, which could affect cancer progression by influencing immune and endocrine function.^3^^,^^4^

The role of personality traits in survival after cancer has been addressed in several prospective studies, but the evidence is limited and no conclusion has been reached. These studies had several limitations, such as small sample size. Almost all had fewer than 200 participants and lacked sufficient statistical power to analyze site-specific cancer. The present author used 2 population-based prospective cohort studies (the Miyagi cohort study^9^ and Finnish twin cohort^6^) to test the hypothesis that personality traits have a role in cancer survival.

First, in July 1990, 41 442 residents of Japan completed the short form of the EPQ-R and a questionnaire on various health habits; 890 incident cases of cancer were identified among the participants between January 1993 and December 1997.^9^ These 890 cases were followed-up until March 2001, and 356 deaths from all causes were identified among them. Cox proportional hazards models were used to estimate HRs for death according to score quartiles for the 4 personality trait subscales, after adjustment for potential confounders. Multivariable HRs for all-cause death among individuals in the highest versus the lowest quartile of personality trait subscale score were 1.0 for extraversion (95% CI = 0.8–1.4; *P* for linear trend = 0.73) and 1.1 for neuroticism (95% CI = 0.8–1.6; *P* for linear trend = 0.24) (Table 2).

**Table 2.** Multivariate-adjusted hazard ratios (HRs) and 95% CIs for all-cause mortality according to score quartile for personality traits subscales among persons with a diagnosis of cancer

Miyagi cohort study^9^ Outcome: Mortality after cancer diagnosis	Exposure	EPQ-R, Extraversion				EPQ-R, Neuroticism			
		
	Score group	Q1 (low)	Q2	Q3	Q4 (high)	Q1 (low)	Q2	Q3	Q4 (high)
		
	Multivariate HR (95% CI)	1.0 (Ref)	0.8 (0.7–1.0)	0.9 (0.8–1.1)	0.9 (0.7–1.1)	1.0 (Ref)	1.0 (0.8–1.2)	1.0 (0.9–1.2)	1.2 (1.0–1.4)
		
	*P* for linear trend	0.32				0.06			
	
	Covariates	Age, sex, smoking, alcohol, body mass index, education, family history of cancer							

Swedish/Finnish twin cohort^6^ Outcome: Mortality after cancer diagnosis	EPI subscales	EPI, Extraversion				EPI, Neuroticism			
		
	Score group	Continuous variables				Continuous variables			
		
	Multivariate HR (95% CI)	1.00 (0.98–1.02)				1.00 (0.98–1.02)			
		
	*P* for linear trend	0.86				0.61			
	
	Covariates	Age, sex, smoking, alcohol, body mass index, education							

Abbreviations: EPG-R, Eysenck Personality Questionnaire–Revised; EPI, Eysenck Personality Inventory; Q, quartile.

Scores on personality subscales were divided into 4 categories to yield quartiles.

The second large prospective population-based cohort study comprised 31 145 Finnish (baseline investigation period = 1976–2004) participants who had completed a questionnaire including the EPI and items on health behavior at baseline.^6^ A total of 2733 cancer cases were identified during a maximum follow-up period of 30 years, among whom there were 1548 deaths during a maximum follow-up period of 29 years. Cox proportional hazards models were used to estimate HRs for all-cause death. HRs were estimated by treating scores on personality traits subscales as continuous variables and are presented as risk per 1-unit increase in score for each scale. In multivariate analyses, extraversion and neuroticism were not significantly associated with all-cause death: the HRs were 1.00 for extraversion (95% CI = 0.98–1.02; *P* for linear trend = 0.86) and 1.00 for neuroticism (95% CI = 0.98–1.02; *P* for linear trend = 0.61) (Table 2).

The data from these Japanese and Finnish population-based prospective cohort studies do not support the hypothesis that personality traits are associated with cancer survival.

## DEPRESSION AND SURVIVAL AFTER CANCER

Negative psychological states, including depression, are common among cancer patients. Between 8%^10^ and 44%^11^ of patients with lung cancer were reported to have depression. Furthermore, it has been suggested that depression affects prognosis and quality of life among patients with lung cancer. In a recent review of studies on associations between depression and mortality risk among cancer patients, depression was associated with mortality risk.^12^ It has been hypothesized that depression affects mortality risk in cancer patients through endocrine and/or immunologic pathways^13^^,^^14^ or through poor compliance with cancer treatment.^15^ Another possible explanation for the increased mortality observed among cancer patients with depression is that depression may simply reflect poor clinical status, which by itself would be associated with increased cancer mortality. Depression was reported to be strongly associated with poor clinical status, as indicated by tumor stage, performance status (PS), and severity of clinical symptoms. In addition, severity of clinical symptoms such as pain and dyspnea was an important independent prognostic factor in a population that included patients with lung cancer. Indices of clinical status are thus important confounders when evaluating the association between depression and cancer mortality. The present study tested the hypothesis that the association between depression and cancer survival among patients with lung cancer is confounded by the poor clinical status of patients.^16^

The author conducted a prospective cohort study using data from the Lung Cancer Database Project at the National Cancer Center Hospital East in Japan.^16^^,^^17^ Between July 1999 and July 2004, 1178 patients with lung cancer were enrolled. The questionnaire included items on socioeconomic characteristics, smoking status, clinical symptoms, and psychological status after diagnosis. Depression status among patients with lung cancer was assessed by using the Hospital Anxiety and Depression Scale. Information on clinical stage, PS, and histological type was obtained from medical charts. The participants were followed-up until December 2004, and 686 died. A Cox regression model was used to estimate HRs for all-cause death. Adjustment for the effects of socioeconomic variables and smoking status (model 1) and for the effects of clinical stage and PS as indicators of clinical status (model 2) did not alter the significant positive association between depression and mortality (*P* for linear trend < 0.001 and 0.04, respectively). However, when self-reported pain and dyspnea were included in the multivariate model (model 3), the association became nonsignificant (*P* for linear trend = 0.26) (Table 3). Multivariate logistic regression analysis using a cross-sectional design to examine the association between indicators of clinical status and depression among study patients showed that more-advanced clinical stage and poorer PS were significantly associated with higher prevalence of depression. Further, more-severe pain and dyspnea were significantly associated with higher prevalence of depression, independent of clinical stage or PS.

**Table 3.** Multivariate-adjusted hazard ratios (HRs) and 95% CIs for all-cause mortality according to score quartile for depression subscale among patients with a diagnosis of lung cancer

Lung Cancer Database Project, Japan^16^ Outcome: Mortality after cancer diagnosis	Exposure	HADS, Depression											
	
	Score group	Q1 (low)	Q2	Q3	Q4 (high)	Q1 (low)	Q2	Q3	Q4 (high)	Q1 (low)	Q2	Q3	Q4 (high)
			
	Multivariate HR (95% CI)	1.0 (Ref)	1.3 (1.0–1.6)	1.4 (1.1–1.7)	1.8 (1.5–2.3)	1.0 (Ref)	1.0 (0.8–1.2)	1.0 (0.8–1.3)	1.3 (1.0–1.6)	1.0 (Ref)	1.0 (0.8–1.2)	1.0 (0.8–1.3)	1.2 (0.9–1.4)
			
	*P* for linear trend	<0.001				0.04				0.26			
			
	Covariates	Model 1: Age at diagnosis, sex, histologic type, education, marital status, smoking				Model 2: Age at diagnosis, sex, histologic type, education, marital status, smoking, clinical status, PS				Model 3: Age at diagnosis, sex, histologic type, education, marital status, smoking, clinical status, PS, self-reported pain, dyspnea			

Abbreviations: HADS, Hospital Anxiety and Depression Scale; PS, performance status, Q, quartile.

Scores on personality subscales were divided into 4 categories to yield quartiles.

The present findings indicate that the association between depression and mortality risk among patients with lung cancer was largely confounded by indicators of clinical status, including clinical stage, PS, and clinical symptoms.^16^

## RISK OF DEPRESSION AMONG PARTNERS OF CANCER PATIENTS

Understanding of the psychosocial consequences of cancer has increased during the past few decades. In response, supportive psychosocial intervention strategies have been developed and are tailored to the problems that cancer patients face during the course of their disease. The extent to which cancer affects patients and their closest relatives was first addressed in a seminal article published more than 20 years ago. House et al illustrated how several diseases affected people close to the patient.^18^ The mechanisms of these effects may involve several interacting pathways: the event may cause stress in the partner; it may deprive the partner of emotional, social, and economic support; and it can influence the daily life and behavior of the partner.^18^^,^^19^ The effect of cancer on the psychological well-being of partners could increase the risk of several psychiatric disorders related to stressful life events, including neurotic, stress-related, somatoform, substance abuse-related, and affective disorders.

The few small studies that have been published thus far suggest that serious psychosocial problems develop among partners of cancer patients; however, to the best of the author’s knowledge, no studies have addressed the risk of severe depression.

A retrospective cohort study of male partners of women with breast cancer used unbiased nationwide population-based data to investigate their risk of hospitalization for an affective disorder.^20^ The study followed 1 162 596 men born during 1925–1973 who were older than 30 years at the time of the study, resided in Denmark between 1994 and 2006, had no history of hospitalization for an affective disorder, and had continuously lived with the same partner for at least 5 years. The Cox regression analysis included detailed clinical information on the diagnosis and treatment of breast cancer and on annually updated socioeconomic and health-related data obtained from national administrative and disease registers. During the 13-year follow-up period, breast cancer was diagnosed in the partners of 20 538 men. In multivariable analysis, men whose partner was diagnosed with breast cancer were at increased risk of being hospitalized with an affective disorder (HR = 1.39, 95% CI = 1.20–1.61, *P* < 0.001; Table 4), and there was a dose–response relation between breast cancer severity and risk of hospitalization. Furthermore, the risk of hospitalization for an affective disorder among men whose partner had died after a breast cancer diagnosis was 3.6-fold that of men whose partner had survived breast cancer.

**Table 4.** Multivariate-adjusted hazard ratios (HRs) and 95% CIs for affective disorder among partners of a woman with breast cancer

Nationwide study, Denmark^20^ Outcome: Hospitalization for an affective disorder	Exposure	Breast cancer diagnosis in partner

	Multivariate HR (95% CI)	1.39 (1.20–1.61)

	*P* value	<0.01

	Covariates	Number of children, highest attained educational level, disposable household income, affiliation with labor market, Charlson index, history of alcohol-related mental disorders

## CONCLUSIONS AND FUTURE IMPLICATIONS

### Personality traits and cancer risk

The association between neuroticism and prevalent cancer may be a consequence rather than a cause of cancer diagnosis and symptoms. Further, although residual confounding may never be totally eliminated, the present findings strongly suggest that the overall effect size for a causal association between personality and cancer is extremely small. Thus, such an association, if it exists at all, is unlikely to have clinical or public health implications.

### Personality traits and survival after cancer

Personality traits such as extraversion and neuroticism are not direct risk factors for survival after cancer.

### Depression and survival after cancer

The association between depression and mortality risk among patients with lung cancer was largely confounded by indicators of clinical status, including clinical stage, PS, and clinical symptoms.

### Risk of depression among partners of cancer patients

Men whose partner had breast cancer were at increased risk of hospitalization for an affective disorder. Screening for depressive symptoms among partners and family members of cancer patients is therefore an important issue for research in psycho-oncology.

## ONLINE ONLY MATERIALS

Abstract in Japanese.
