# Supplementary material for: Effect of Psychosocial Factors on Cancer Risk and Survival
Source: J Epidemiol. 2014 Jan 5;24(1):1–6. doi: 10.2188/jea.JE20130124 (PMC3872518; doi:10.2188/jea.JE20130124)
Supplement: Abstract in Japanese. [file je-24-001-s001.pdf]

がんに影響を及ぼす心理社会的要因の検討

中谷直樹
東北大学東北メディカル・メガバンク機構予防医学・疫学部門

パーソナリティ、抑うつなどの心理社会的要因は、免疫機能・内分泌機能を変化させ、がんの発症やがん予後に影響する可能性が指摘されている。これら心理社会的要因とがんの発症やがん予後の関連は多く報告されているが、未だ結論は得られていない。著者らは、一般地域住民を対象としたデータベース及び臨床データベースを用いて上記の関連を検討した。結果として、パーソナリティ、抑うつなどの心理社会的要因ががんの発症やがん予後と関連せず、仮説は否定された。

一方、最近の報告において、がん患者のみならずその配偶者・家族の健康影響が指摘されている。配偶者・家族の健康影響のメカニズムとして、がん患者への介護や死別による心理的・身体的負担、がん患者と共通する不健康な生活習慣が考えられる。先行研究において、がん患者の配偶者は重大な心理社会的問題を生じることが報告されているが、研究デザイン・研究規模などの問題があった。著者らは、デンマークの国家規模のデータベースを利用して、乳がん患者の男性パートナーのうつ病リスクを後ろ向きコホート調査にて検証した。結果として、男性パートナーのうつ病リスクは有意に増大し、仮説は支持された。

これまでの著者らの研究による結論として、パーソナリティ、抑うつなどの心理社会的要因とがんの発症やがん予後の関連はない、あるいは極めて小さいことが示された。一方、がん患者のパートナーのうつ病リスクは増大することが示された。今後、サイコオンコロジー分野において、がん患者のパートナー・家族の抑うつ症状のスクリーニングを実施し、早期発見することの重要性が明らかとなった。

キーワード：がん、コホート研究、抑うつ、パーソナリティ、心理社会的要因、リスク、生存
